# Infection and Immune Memory: Variables in Robust Protection by Vaccines Against SARS-CoV-2

**DOI:** 10.3389/fimmu.2021.660019

**Published:** 2021-05-11

**Authors:** Pankaj Ahluwalia, Kumar Vaibhav, Meenakshi Ahluwalia, Ashis K. Mondal, Nikhil Sahajpal, Amyn M. Rojiani, Ravindra Kolhe

**Affiliations:** ^1^ Department of Pathology, Medical College of Georgia, Augusta University, Augusta, GA, United States; ^2^ Department of Neurosurgery, Augusta University, Augusta, GA, United States

**Keywords:** SARS–CoV-2, Pathogen, Immune system, Immune memory, Vaccine, Vaccine design, Immunological memory, T cells

## Abstract

SARS-CoV-2 is the cause of a recent pandemic that has led to more than 3 million deaths worldwide. Most individuals are asymptomatic or display mild symptoms, which raises an inherent question as to how does the immune response differs from patients manifesting severe disease? During the initial phase of infection, dysregulated effector immune cells such as neutrophils, macrophages, monocytes, megakaryocytes, basophils, eosinophils, erythroid progenitor cells, and Th17 cells can alter the trajectory of an infected patient to severe disease. On the other hand, properly functioning CD4+, CD8+ cells, NK cells, and DCs reduce the disease severity. Detailed understanding of the immune response of convalescent individuals transitioning from the effector phase to the immunogenic memory phase can provide vital clues to understanding essential variables to assess vaccine-induced protection. Although neutralizing antibodies can wane over time, long-lasting B and T memory cells can persist in recovered individuals. The natural immunological memory captures the diverse repertoire of SARS-CoV-2 epitopes after natural infection whereas, currently approved vaccines are based on a single epitope, spike protein. It is essential to understand the nature of the immune response to natural infection to better identify ‘correlates of protection’ against this disease. This article discusses recent findings regarding immune response against natural infection to SARS-CoV-2 and the nature of immunogenic memory. More precise knowledge of the acute phase of immune response and its transition to immunological memory will contribute to the future design of vaccines and the identification of variables essential to maintain immune protection across diverse populations.

## SARS-CoV-2 and the Acute Phase of Infection

Severe acute respiratory syndrome coronavirus 2 (SARS-CoV-2) is a novel strain of coronavirus responsible for the current pandemic that has infected more than 140 million and caused the death of more than 3 million individuals globally (https://coronavirus.jhu.edu/). Among infected individuals, 80% of patients display mild symptoms or are asymptomatic, 15% required oxygen (O_2_) and about 5% have critical pneumonia-like symptoms and require assisted ventilation (1). As more than a year has passed since the origin of this pathogen, there is tremendous interest in the long-term properties of immunological memory of recovered individuals as it can assist in design & improvement of next generation vaccines (2). As of March 2021, there are currently 8 vaccines approved for full use and 5 vaccines in early or limited use (3). Among these, the first two authorized vaccines were modified mRNA vaccines by Pfizer-BioNTech (Tozinameran) and Moderna (mRNA-1273). These two vaccines were predominantly rolled out in high-income countries and require ultra-cold chain infrastructure (-70°C for Pfizer vaccine). Other approved vaccines exhibit fewer logistic challenges and are suited for medium to low-income countries where they can be transported and stored in standard 2-8°C conditions (4). Among the most populous countries in the world, India and China have ramped up immunization efforts using their indigenous vaccines developed by Bharat Biotech and CanSino Biologics respectively. Further, there are 23 vaccine candidates in Phase 3 trials and its hoped that millions of individuals would be vaccinated in the next few months globally (3). Intensive research is underway to understand similarities and variations in the immune response in naturally recovered patients and vaccine-induced immunization. In this review, we will discuss the natural immune response to SARS-CoV-2 with particular emphasis on immunological memory.

The SARS-CoV-2 (+) RNA genome is known to encode 29 proteins (5, 6). The protein repertoire includes structural proteins: spike (S), membrane (M), envelope (E), nucleocapsid (N), and 16 nonstructural proteins (NSP 1-16) (7). Additionally, there are 9 accessory proteins (ORFs - 3a, 3b, 6, 7a, 7b, 8, 9b, 9c, 10) (8). Although main structural proteins and NSPs are studied in considerable detail, accessory proteins are emerging as important mediators of SARS-CoV-2 pathophysiology. In a recent study, accessory protein ORF9b was found to promote infection by binding to a mitochondrial chaperone protein named Tom70 (9).

The entry of the virus into the cell requires the presence of two host proteins, ACE2 and TMPRSS2. ACE2 acts as an entry receptor while TMPRSS2 acts as a cellular protease for priming of viral spike protein which is required for fusion with the host cell membrane (10). Recently, the expression of ACE2 and TMPRSS2 has been confirmed in salivary glands and oral mucosa epithelia which implicates the role of the oral cavity and saliva in the transmission of SARS-CoV-2 (11). In respiratory tissues, co-expression of ACE2 and TMPRSS2 is only restricted to type II pneumocytes and a subset of epithelial cells. Ziegler et al. reported that respiratory tissue shows a poor expression of ACE2 and TMPRSS2 with only 0.8% of type II pneumocytes with co-expression of both the proteins (12). Interestingly, SARS-CoV-2 has more than 10-20-fold higher affinity for ACE2 compared to other coronaviruses, and it is purposed as one of the several reasons for its harsh pathophysiology (13). Meanwhile, the expression of ACE2 has also been documented in two other cells: enterocytes of the small intestine and goblet secretory cells of the nasal mucosa (12). These findings hint at the presence of other receptors/pathways which can be used by the virus to infect the host cells. Recently, two studies have identified Neuropilin-1 as another novel cell surface receptor for the entry of SARS-CoV-2 (14, 15). In addition to these receptors, the role of other co-receptors such as sialic acids, heparan sulfate, CD147, or GRP78 needs to be investigated in greater detail as they can provide novel opportunities to design therapeutic interventions against COVID-19 (15, 16).

The SARS-CoV-2 infection leads to broad activation of innate and adaptive limbs of the immune system in humans. In the adaptive immune system, T cells play a critical role in managing the immune response to the viral pathogens (17). For its activation, T cells depend on the interaction between TCR (T Cell Receptor) and peptide-MHC complex (Major Histocompatibility Complex also known as HLA - Human Leukocyte Antigen in humans). One of the important variables to be considered in this interaction is the effect of genetic polymorphism in the process of antigen presentation and its association with the risk of COVID-19 (18). Individuals with specific allele, particularly HLA-B*46:01 are found to be pre-disposed to severe form of COVID-19 disease. On the other hand, individuals with HLA-B*15:03 had higher capacity to present SARS-CoV-2 epitopes to T cells and were associated with mild COVID-19 symptoms (19, 20). Although further studies are required to establish this correlation in diverse populations, it has been proposed that individuals with high-risk HLA alleles could be prioritized for vaccination (20).

**Figure 1:**
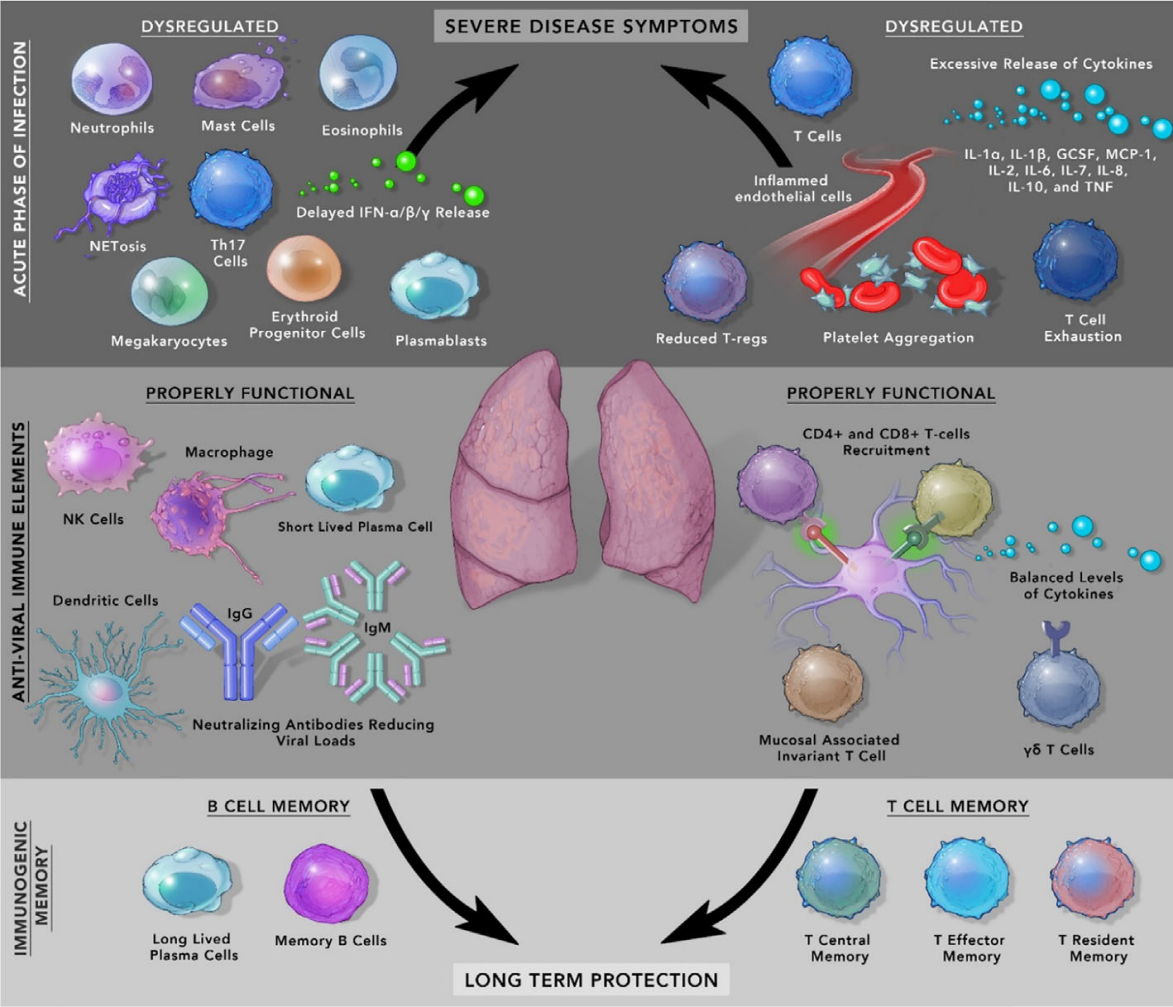
Mediators of Immunopathology in SARS-CoV-2 infection and resolution.

At the clinical level, COVID-19 patients show diverse symptoms, ranging from mild to life-threatening acute respiratory distress syndrome (ARDS) which were also present during MERS and SARS-CoV-1 infection (21). The accumulation of pro-inflammatory cytokines like IL-1α, IL-1β, IL-17A, IL-12 p70, IFN-α, IFN-γ, TNFα and other cytokines included in the ‘cytokine release syndrome’ increases proportionally with an increase in viral load (22). In some patients with comorbidities, symptoms can be severe from septic shock, multi-organ failure due to capillary leaks, to the formation of thromboemboli and organ dysfunction. Another important variable in the form of sex difference has emerged, which has resulted in males developing a higher risk of mortality and severe illness compared to females. It is likely due to inherent immunological differences between males and females. One of the reasons for this difference could be higher levels of baseline pro-inflammatory cytokine, chemokines, and the presence of improved T-cell activation in females (23). In circulation, lower numbers of blood lymphocytes characterized as Lymphopenia is a prognostic indicator in COVID-19 patients as its level drops drastically in patients with severe disease whereas, moderately ill patients showed less fluctuation (24) Besides, rise in neutrophils, low lymphocyte-to-CRP and low neutrophil-to-lymphocyte ratio (NLR) have emerged as indicators of severe COVID-19 progression (25, 26). Several other immune cells play critical roles in COVID-19 patients and are shown in **Figure 1** and **Table 1**.

**Table 1:** The role of immune cells in inflammation, homeostasis, and SARS-CoV-2 pathophysiology.

Innate immune cells		
**Immune cells**	**Immune cell function in inflammation and homeostasis**	**Emerging clinical relevance in SARS-CoV-2 infection**
Neutrophils	Neutrophils are first responders at the site of infection and contribute to acute lung injury (27). Apart from its role in inflammation, apoptosis of neutrophils serves as a signal for withdrawal of tissue damage (28).	Neutrophil responsive chemokine signature, secretion of NET (Neutrophil extra-cellular traps), and increased infiltration of neutrophils were found to be associated with severe cases of COVID-19 (29–32).
Mast cells	Mast cells with poor regulation of pre-formed inflammatory granules can lead to severe pathology of the lungs (33). In addition to inflammatory function, mast cells can contribute to homeostatic functions through the secretion of anti-inflammatory cytokines and wound healing processes (34).	Dysfunctional mast cells and release of histamines leads to hyperinflammation hyperinflammatory cytokine storm in COVID 19 patients with severe disease (35, 36).
Basophils	Basophils are similar in function to mast cells and release pre-formed mediators upon IgE-induced activation (37, 38). Basophils in the lungs have been shown to maintain lung homeostasis by regulating the maturation and function of alveolar macrophages (39).	Basophils are reduced in the acute phase but increase in the recovery phase. Basophils were found to enhance B cell response and production of strong IgG antibody titers (40).
Eosinophils	Eosinophils can exacerbate tissue damage by contributing inflammatory cytokines and lipid mediators (38). In normal conditions, eosinophils play several roles including glucose homeostasis, immunomodulation, and other biological functions (41).	IFN-γ triggered expansion of CD62L+ Eosinophils contributes to ARDS. Eosinophil levels were found to increase in the recovery phase of COVID-19 patients (40).
Dendritic cells	Airborne pathogens and debris are removed by lung-resident dendritic cells. These cells cross-present antigens to naïve T cells after migrating to lymph nodes to activate immune response (42).	Impaired functionality of dendritic cells was found in SARS-CoV-2 infected patients (43).
Monocytes	Monocytes along with granulocytes have been shown to emigrate to naïve tissues for maintenance of normal tissue functions (44). In diseased conditions, pulmonary monocytes can initiate and activate CD8+ T cells in the lungs during infection (45).	SARS-CoV-2 induces mixed M1/M2 phenotype in circulating monocytes (46).
Macrophages	Macrophages contribute the majority of cellular immune content in homeostatic lungs and are composed of three subtypes: bronchial macrophages, interstitial macrophages, and alveolar macrophages (42).	Patients with higher viral load demonstrated T cell exhaustion and correlated with CCL15 expressing M1-like macrophages (47).
**Adaptive immune cells**		
B cells	Among all immunoglobulins, IgA is the most prevalent in the lungs and is secreted by B cells and plasma cells (48).	A reduced number of ‘Naturally effector’ B cells were found in COVID-19 patients (49).
Plasmablasts	Plasmablasts mature into plasma cells that secrete IgA, IgM, IgD, IgG, and IgE, essential for contributions to the health and disease of lungs (48).	PBs showed metabolic shift to higher amino-acid metabolic pathways in severe patients which is reduced in convalescent-phase (50).
CD4 T cells	Naïve T cells can differentiate into effector or memory T cells upon exposure to antigen through antigen-presenting cells (APCs) (51).	SARS-CoV-2 infected patients showed TH1 cytokine profile (52).
CD8 T cells	CD8+ T cells produce IFN-γ, TNF-α, and IL-2, which leads to the killing of infected cells using cytotoxic granules (granzyme and perforin) (51).	Decrease in CD8+ T cells in severe cases (32).
T memory cells	T resident memory cells are present in the lungs for rapid control of respiratory viral infections (53).	Long-lasting T cell immunity was found to be present in COVID-19 recovered patients (54).
B memory cells	Resident memory B cells play a significant role in the adaptive immunity of lungs (55).	B memory cell response persists after the recovery phase (56).
T-regulatory cells	T regulatory cells in the lungs promote tolerance to inhaled antigens and prevent excessive inflammation (57).	Reduction of T-reg cells was observed in severe to moderate COVID-19 patients (58–60).
**Other immune cells**		
Monocytic myeloid-derived suppressive cells (M-MDSCs)	MDSCs are present in pathological conditions such as infection or cancer (61).	Higher frequency of M-MDSCs in acute patients (43).
Polymorphonuclear (PMN)-MDSC		Expansion of PMN-MDSCs correlated with ICU patients and inflammatory cytokines: IL-1β, IL-6, IL-8, and TNF (62).
NK cells	NK cells provide immunity against viral infections through antibody-dependent cellular cytotoxicity and cytotoxic lysis (63). In steady conditions, lung NK cells are predominantly in the hypofunctional state to prevent unwanted, excessive inflammation (63).	Lowered NK cells and effector functionality (64).
NK memory cells	Memory-like NK cells with robust recall properties can play a vital role during viral infection (65).	A significantly higher number of memory NK cells in deceased patients (66).
Innate lymphoid cells	During infection, Innate lymphoid cells play a critical role in the repair of mucosal surfaces (67). After infection, these cells promote pulmonary homeostasis through mechanisms such as wound healing and upregulation of amphiregulin (68).	Severe patients had a lower frequency of ILCs (69).
Gamma delta T cells (γδT cells)	γδT cells have both innate and adaptive features for protection against invading pathogens (70).	Depleted levels of γδT cells were found in severe patients (49).
Mucosa-associated invariant T cells (MAIT cells)	MAIT cells are activated by conserved pathogenic ligands and play a protective role (71).	MAIT cells are actively recruited to inflamed airways of COIVD-19 patients. There was a significant reduction in MAIT cells in severe COVID-19 patients (69, 72).
TH17 cells	TH17 inhibits Th1 type immune response and can contribute to immunopathology during viral infections (73). In a steady state, IL-17A plays an important role in the repair and maintenance of epithelial cell homeostasis (74).	TH17 activation has been associated with severe COVID-19 symptoms (75).

In addition to understanding the immune response from circulating blood, two other sources with much more information from the site of infection are Bronchoalveolar lavage fluid (BALF) and autopsy specimen. BALF analysis has identified increased infiltration of macrophages in patients with severe disease compared to moderately ill patients. CD8+ T cells in severe patients showed higher proliferation, energy production, and translation capacity whereas, patients with moderate symptoms had T-cells in the proliferative stage (76). In another study, single-cell analysis identified pro-inflammatory macrophages at higher levels in severe patients compared to clonally expanded CD8+ T cells (77). Lung autopsy of COVID-19 patients has shown 2 distinct categories of interferon-stimulated genes (ISGs), ISG^high^ (high viral load and pro-inflammatory cytokines) with early mortality compared to ISG^low^ with low viral load. Interestingly, lung morphology was relatively intact in ISG^high^ patients compared to ISG^low^ patients which showed significant lung damage. In this study, activated T cell signature was found in ISG^low^ patients with a low viral count which might be indicative of their importance in antiviral immunity (78).

## SARS-CoV-2 and the Immunity Phase

When infected by respiratory pathogens, innate and adaptive immune cells eliminate the pathogen and lead to formation of memory immune cells for rapid immune response against future infection. There is evidence of protective immunity conferred by B-cells in SARS-CoV-2 infected patients. The spike and nucleocapsid proteins specific antibodies are detectable as early as 6 days after the confirmation of COVID-19 (79, 80). It has been shown that the presence of neutralizing titers of IgG antibodies against SARS-CoV-2 can offer protection against re-infection (81). There is emerging evidence of potent memory B cell response in COVID-19 recovered individuals (82). It has been additionally shown in longitudinal studies of mildly infected patients, that while the initial spike in IgA antibodies drops significantly, the levels of IgG antibodies remain elevated for duration of at least first 3 months (83). In a recent study, IgG antibodies against spike proteins were found to be stably produced for over 6+ months post-infection. SARS-CoV-2 CD4+ T cells and CD8+ T cells showed a half-life of 3-5 months (84). A recent comparative study between severe and mild patients showed better humoral immunity in severe patients because of a higher level of BCR clonal expansion and activation (85). In contrast to these observations, Gattinger et al. used inhibition assay and found that S- (Spike) specific and RBD- (Receptor-Binding Domain) specific antibodies did not always correlate with inhibition of RBD binding to ACE2. In this small study, 25 convalescent patients showed a range of inhibition levels, from complete lack of inhibition to even enhanced RBD binding in some of the subjects (86). It should be pointed out that natural SARS-CoV-2 infection does not lead to protective antibody response in all patients. These factors should be carefully assessed while quantifying the efficacy of vaccine-induced immune memory.

In addition to the humoral response, SARS-CoV-2 infection elicits protective immunity as shown by the persistence of B_M_ (Memory B cells) and T_M_ (Memory T cells) in recovered patients (87–89). The resolution phase after an infection is followed by the death of most of the immune cells except around 10% ‘long-lived memory cells’ (90). These memory cells can provide long-term robust protection against future infections. Interestingly, the transition of IgM+ and IgG+ B_M_ generates a transitionary bi-phasic appearance during the post-infection period. IgM+ B_M_ cells were found to be present during the initial 20 days of infection up to 150 days, which was followed by IgG+ antibodies (84). Further, there has been evidence of a decline in circulating virus-specific antibodies against spike protein, NCP, or non-structural proteins (91–93). Despite this drop, a numerically significant number of RBD- and NCP-specific B_M_ cells remain in circulation as much as 240 days after onset of symptoms (94). However, most of the RBD- B_M_ cells showed CD27+ phenotype compared to NCP- B_M_ cells which suggest that RBD- cells have undergone more cell divisions with higher number of somatic hypermutations (94) (95). It has been shown that B_M_ transitions through phases, as antigen-exposed B_M_ cells demonstrate multiple replication cycles, class switching, and somatic hypermutation (95) (96). CD21^low^ CD27+ activated B_M_ cells are present for a short duration for about 2 weeks and are followed by resting B_M_ expressing CD21+ CD27- (97, 98). The presence of TAC1, CD80, CD180 cells shows that they can activate quickly when exposed to infection again (95, 99). SARS-CoV-2 studies have shown robust B cell memory response as long as 8 months after the start of COVID-19 symptoms (84). Further, these B_M_ cells have been found to correlate positively with T follicular helper cells (Tfh) cells which suggests increased germinal center activity (94). Tfh cells help B cells in affinity maturation and are essential for the generation of memory plasma cells (100). This study has shown that although antibody numbers are reduced over time, the memory B cells compensate for this drop and are ready for active deployment. Some researchers have proposed the identification of these B_M_ cells to be better predictors of long-term immunity compared to antibodies (94).

When infected by respiratory pathogens, the innate, and cellular components of the adaptive immune system function in succession to eliminate and generate immunogenic memory against the pathogen (101). The humoral immune response to SARS-CoV-2 is short-lived which puts specific focus on T cell immune memory. In comparison, B cell response to SARS-CoV-1 was also short-lived but virus-specific CD8+ T cells have been shown to persist for 6-11 years (102, 103). In another study, memory T cells against SARS-CoV-1 were detected even 17 years after the previous pandemic, hinting at the long-term immune response against the pathogens (17). In a recent study, almost half of donor blood samples between 2015 and 2018 showed reactivity to SARS-CoV-2, well before the emergence of this pathogen in the human population. It is speculated that this might be due to the cross-reactivity of other common cold coronaviruses with SARS-CoV-2 (104). This type of ‘heterologous immunity’ is from peptides of diverse viruses and is considered a widespread phenomenon but its role in protection against SARS-CoV-2 needs to be investigated.

There has been accumulating evidence that SARS-CoV-2 memory T cells are produced and maintained in individuals recovered from illness. The response of T cells against a range of SARS-CoV-2 epitopes such as M, N, and 6 other ORFs along with spike protein has been identified. CD4+ T cell response against SARS-CoV-2 spike protein was found to correlate with levels of IgG and IgA titers (105). Further, in COVID-19 patients, a positive correlation of lymphopenia with disease severity was found. However, the qualitative response of CD4+ T cells was found to be impaired in critically ill patients as they had a lower number of virus-specific CD4+ T cells with decreased IL-21, IL-4, and IFN-γ production (106). In one study, polyfunctional Th1-specific response with the secretion of IFN-γ, TNF-α, IL-2 was seen in patients with mild symptoms whereas, it was found to shift toward Th2 in ICU patients (106). Additionally, a higher frequency of Th17 cells and release of IL-17 was found to be associated with severe cases of COVID-19 (75). In contrast, another study found a lower distribution of Th1, TH17 compared to a higher percentage of Th2 cells in COVID-19 patients. Interestingly, this study also found an increase in senescent Th2 cells in patients who died from COVID-19 (107). In a different post-mortem study on severe COVID patients, lymph and spleen analysis revealed a lack of germinal centres, an increase in Th1 cells and a decrease in Th2 cells (108). Additionally, allergic diseases and asthma with its characteristic type-2 inflammation might not be a risk factor for SARS-CoV-2 infection. Apart from IL-4 and IL-5, IL-13 has been shown to reduce the expression of ACE-2 (109, 110). Asthma patients have been broadly divided into Type-2 asthma and Non-Type 2 asthma with different inflammatory profiles and resulting risk susceptibility. While T2 asthma patients elicit a strong TH2 response and possess lower COVID risk, Non-T2 asthma patients express higher Th1 response and COVID disease severity with pre-dominant lung destruction (111). In another interesting study, *ex vivo* analysis of IL-13 reduced expression of both ACE2 and TMPRSS2 in airway epithelial cells. Further, this study also found higher expression of TMPRSS2 but lower expression of ACE2 in nasal epithelial cells of type 2 asthma and allergy patients (112). The influence of the Th1/Th2 paradigm and its relationship to SARS-CoV-2 infection and disease severity needs to be characterized in greater detail.

There are several other SARS-CoV-2 induced variations at the cellular and metabolic level that play an essential role in shaping the immune response of humans. Apart from the reduction of CD4+T cells, lower number of NK cells and an increase in inflammatory monocytes has been observed in COVID 19 patients (50, 113). In another study, similar findings in severe patients have been reported along with the downregulation of HLA class II, increase in inflammatory cytokines, and neutrophil count (114). At the cellular level, an epigenetic modification was observed with hypomethylation of genes corresponding to innate immune signaling, the release of IL-1β, and TNF-α. Comparatively, genes of ATP metabolism and T cell receptor signaling showed decreased expression (50). Metabolic exhaustion of immune cells has also shown to play a vital role in COVID-19 pathogenesis. The activation process of lymphocytes involves switching between glycolysis and mitochondrial respiration that require additional mitochondrial biogenesis, protein translocation, and glycosylation pathways (115). In COVID-19 patients, Plasmoblasts (PBs) are shown to be metabolically active with the excessive shuttling of glucose for antibody glycosylation that might lead to metabolic exhaustion of these cells (116, 117). Further, if exacerbated, alteration in antibody formation such as the formation of fucosylated-IgG antibodies might lead to critical illness in COVID 19 patients (118). In another study, increase in the frequency of PBs was found in severe patients that normalized after sickness (50, 119). Further, Megakaryocytes (MKs) were found to be significantly high characterized by high Interferon expression signature (50). In the autopsy analysis of COVID 19 patients, MKs and thrombosis were observed in several organs including a rare finding of platelet-derived microthrombi in the heart (120). Systematic multi-organ complications might be facilitated by these damaging interactions of immune cells and tissue. Critically ill patients with poor oxygen circulation have shown an increase in numbers of Erythroid progenitor cells which derive from bone marrow due to hypoxic stress. The upregulation of GATA1 through HIF1 regulation leads to an increase in circulation of these cells and has been linked to heightened immune response (50, 121). The emerging role of epigenetic regulation and metabolic exhaustion in the generation of immunogenic memory should increase our understanding of their participation in immune memory against SARS-CoV-2.

Upon activation, T cells can move along multiple trajectories for clearance of pathogen but the T cells with effector functions die after few days of the onslaught, leaving behind a pool of memory T cells. These T cells can be divided into three broad categories based on their trafficking pattern, anatomical location, and activity: T central memory (T_CM_), T effector memory (T_EM_), and T resident memory (T_RM_) (122). T_EM_ displays rapid generation granzyme and IFN-γ but very low proliferative capacity. T_CM_ on the other hand, with CD62L and CCR7 show homing capacity for secondary lymphoid organs where they can be deployed to prevent systemic infection, activate effector functions, and infiltrate peripheral tissues for rapid response against pathogens. This fits into the biphasic response whereby T_EM_ initial onslaught against pathogen and later new range of T cells from T_CM_ pool gets ready for the final elimination of the pathogen (123). Overall, the difference between effector T cell response and memory T cell response includes an increased pool of memory T cells reactive against the pathogen, pre-programmed specific effector response generates rapid response against a specific pathogen, and generation of resident memory T cells T_RM_ in peripheral tissues (124). Additionally, other memory immune cells are also being identified with their role in SARS-CoV-2 pathogenesis. In a recent study, SARS-CoV-2 specific CD8+ T cells was found to consist of two prominent populations of T_EMRA_ cells (terminally differentiated effector memory cells re‐expressing CD45RA) and T_scm_ (T stem cell memory) cells. T_EMRA_ cells have been identified to play an important in protection against SARS-CoV-2. These SARS-CoV-2 antigen experienced tissue resident CD8+ T cells re-express CD45RA, a naïve cell marker known to offer protection in tissues such as spleen, blood, and lung (125). The role of T_EMRA_ cells was explored in a different study using deep immune profiling analysis that identified three major immunotypes of COVID-19. Immunotype 1 was associated with disease severity, activated CD4+ T cells, low circulatory circulating follicular helper cells, and T_EMRA_ cells. Immunotype 2 group did not show any association with disease severity and showed lower CD4 T cell activation and proliferation memory B cells. Immunotype 3 was characterized by lack of T and B cell response and negative association with disease severity (119). Further, the importance of other immune cells, particularly memory Tfh cells is critical against SARS-CoV-2 as these CD4+ T cells originating from GC-Tfh cells play a central role in the event of re-exposure to the antigen (126). Whether the SARS-CoV-2 long-term immunity can maintain this pool of memory Tfh cells remains to be determined. Cumulatively, these studies show that the complexity of immune memory in SARS-CoV-2 infection will form a critical component while analyzing the long-term efficacy of vaccine-induced memory response to SARS-CoV-2.

There is another critical host factor that plays a central role in shaping the detrimental effect of SARS-CoV-2 infection. “Immunosenescence”, characterized by decline and dysregulation of immune response due to aging plays a significant role in COVID-19 pathogenesis. It includes gradual deterioration in the form of reduced thymus function, chronic stimulation by antigens and corresponding increase in proinflammatory mediators, latent reactivation of pathogens, and poor response to vaccines. This “Inflammaging” also assists in alternative activation of M2-macrophages with a dysregulated phagocytic capacity which can lead to adverse outcomes in many infectious diseases (127) (128). The co-morbidity of heart and lung diseases puts the elderly population at mortality risk from viral infections (129). As the individual grows older, the pool of naïve T cells declines along with reduction of proper environment, a conducive *milieu* in the form of cytokines, cell-cell interaction, and chemokines, which are essential for the proper functioning of the immune response. The lack of a proper environment for their functionality can sometimes lead to an excessive life-threatening situation of a cytokine storm. On the other end, paucity of proper signaling, ineffective priming of T cells by antigen-presenting cells can lead to exhausted T cells with chronic poor effector response.

## SARS-CoV-2 Vaccine and Mediators of Durable Immunity

The greater understanding of the immune response to SARS-CoV-2 is assisting the scientific community in the identification of key variables essential to study the long-term immune response to this novel pathogen. There are several important variables to be considered to understand the durability of vaccine-induced immune memory. To begin with, epigenetic programming of innate cells has shown protection against SARS-CoV-2. In a randomized controlled study, BCG administration showed significant delay in first infection compared to the placebo group (median: 16 months Vs, 11 months respectively) (130). BCG or other vaccines can trigger ‘pathogen-agnostic antimicrobial resistance’ due to increased baseline immunity (131). It has been proposed that these live vaccines (including oral polio and measles) can boost trained immunity through reprogramming of immune cells at epigenetic, transcriptional, and functional levels (132). It should be further explored as to how much ‘trained immunity’ can contribute to protection against infective agents of the pandemic.

Several studies have explored immune variations with protection against SARS-CoV-2 but currently, there is a lack of immune correlate of protection associated with the COVID-19 (133). Currently, nABs (neutralizing antibodies) are considered the benchmark to quantify protection but a decline in production of nABs can be observed in some of the SARS-CoV-2 recovered patients (134). In a recent study, a mechanistic link was found between SARS-CoV-2 infection and resulting B cell response with limited durability. In this study, severe SARS-CoV-2 infected patients showed blunt germinal centers, reduction in Bcl-6 expressing B cells and Tfh cells. Further, the presence of a high amount of TNF can also reduce GC response and block differentiation of Tfh cells necessary for B cell maturation (108). There are other mediators of immunity such as T-cells, that can complement nABs as a potential variable to quantify immune protection. In fact, heterogeneous immunological memory has shown discordance where recovered individuals displayed both ends of the spectrum: High nAB/low T cell and low nAB/high T cell (135). In another study, 93% exposed asymptomatic individuals mounted T cell while only 60% showed seropositive status (136) On the other hand, T cells have shown reactivity even in the absence of antibodies in asymptomatic individuals thus, highlighting the potent role of memory T cells in COVID-19 infection (88). Further, CD8+ T cells of recovered individuals showed higher expression of TCF1 and can quickly differentiate into diverse T cells such as Tfh in case of re-exposure to SARS-CoV-2 (88). It has been speculated that the presence of other means of protection such as T_RM_ can lower the threshold of nAB required for protection against COVID-19. It can cause rapid deployment of effector T cells and other leukocytes to provide synergistic protection. T_RM_ can also directly influence the production of antibodies (137, 138). Further, T cell activation and resulting reduction of Treg cells can also play a critical role in maintaining the immunity in T cell patients. T cells are known to express activation markers such as CD38, HLA-DR, and Ki67 (54). It has been observed that the exhaustion markers such as programmed cell death marker 1 (PD-1) and the receptor mucin domain-containing protein-3 (TIM-3) leads to the poor effector function of T cells (139). Interestingly, T cell hyperactivation fuels its exhaustion which leads to a reduction in the activity of T-reg cells. In fact, circulating levels of Treg cells were found to be reduced in severe patients compared to COVID-19 with mild symptoms (58, 60). It would be interesting to explore the relative contribution of reduction of T-reg cells in SARS-CoV-2 induced pathogenesis.

The activation of T cells requires interaction between T cell receptor and antigen-MHC complex I (Human leukocyte antigen – HLA in humans) to induce an immune response against infection. The spike protein is a major T cell epitope in other coronaviruses whereas in SARS-CoV-2, the epitopes are spread out from spike to nucleocapsid and matrix protein which results in multiple co-dominant CD4 T cell epitopes (138). Clinically, the magnitude and range of epitope coverage was found to be higher in severe cases compared to mild ones after SARS-CoV-2 infection (140). Further, a higher number of multi-cytokines producing T cells (M/NP) compared to anti-spike CD8+ T cells was found in recovered patients which indicates the importance of other viral proteins as a target for future vaccines (140). Among first approved vaccines, Pfizer, Moderna, and many other candidate vaccines are targeting spike protein (S) but other vaccine candidates are targeting other proteins such as viral protease (Mpro) and RNA-dependent polymerase (RdRp) among others in an effort to induce potent immune response and protection against COVID-19 (141). In another attempt to provide broad immunity by activating multifunctional CD4 T cells, vaccine developer Novavax have incorporated antigens such as M (Matrix protein) as an adjuvant along with standard trimeric SARS-CoV-2 S protein as a vaccine target (142). Apart from these antigens, a recent study has addressed the need for novel antigens by mapping the full landscape of exact antigens involved in SARS-CoV-2 infection. This study recognized a total of 122 immunogenic epitopes in SARS-CoV-2 infected patients among a total of 3141 epitopes. They also found that most immunodominant peptides are located in ORF1 and ORF3 of SARS-CoV-2 genome (143). These observations might help in understanding the quality of memory immune response especially considering that ORF1 is highly conserved among coronaviruses and there is baseline heterologous immunity against other coronaviruses in general population (143). Further, these antigens expand the horizon for vaccine design as it has been speculated that an increase in the number of epitopes may be beneficial in the generation of a more robust CD8+ T cell response (138). It would be interesting to compare and analyze the extent and durability of immune protection provided by vaccines based on single spike protein, other antigen(s) or the use of antigens in the form of adjuvant. In this light, any promising vaccine should define a correlate of protection in the form of durable B and/or T cell response. The unprecedented pace of discovery has led to the development of vaccines in a record time and the continuation of subsequent research is bound to decode the complexity of mediators required for protection in a wider population.

## Author Contributions

PA, RK, KV, and MA drafted the manuscript. AM, NS, and AR provided intellectual inputs. RK supervised the project. All authors contributed to the article and approved the submitted version.

## Funding

KV and RK acknowledges the support provided in the form of start-up grant by Augusta University. The funding bodies had no role in design, analysis, and interpretation pertaining to this study.

## Conflict of Interest

The authors declare that the research was conducted in the absence of any commercial or financial relationships that could be construed as a potential conflict of interest.
